# Nursing Care on HIV/AIDS-Positive Men Who Have Sex with Men: A Qualitative Descriptive Study of Nurse’s Perspective in Indonesia

**DOI:** 10.3390/healthcare10122485

**Published:** 2022-12-08

**Authors:** Kurniawan Kurniawan, Herni Susanti, Mustikasari Mustikasari, Khoirunnisa Khoirunnisa, Nurlaila Fitriani, Iyus Yosep, Efri Widianti, Kusman Ibrahim, Maria Komariah, Sidik Maulana, Hidayat Arifin

**Affiliations:** 1 Department of Mental Health Nursing, Faculty of Nursing, Universitas Padjadjaran, Sumedang 45363, Indonesia; 2 Department of Mental Health Nursing, Faculty of Nursing, Universitas Indonesia, Depok 16424, Indonesia; 3 Department of Pediatric, Faculty of Nursing, Universitas Padjadjaran, Sumedang 45363, Indonesia; 4 Department of Mental Health Nursing, Faculty of Nursing, Universitas Hasanudin, Makassar 90245, Indonesia; 5 Department of Medical-Surgical Nursing, Faculty of Nursing, Universitas Padjadjaran, Sumedang 45363, Indonesia; 6 Department of Fundamental Nursing, Faculty of Nursing, Universitas Padjadjaran, Sumedang 45363, Indonesia; 7 Professional Nursing Program, Faculty of Nursing, Universitas Padjadjaran, Sumedang 45363, Indonesia; 8 School of Nursing, College of Nursing, Taipei Medical University, Taipei 110, Taiwan

**Keywords:** HIV/AIDS, men who have had sex with men, sexual and gender minorities, nursing care, perception

## Abstract

HIV/AIDS-positive men who have had sex with men (MSM) account for roughly one-third of new infections in the region, with numerous nations facing a high and rising prevalence. They often face stigmatization and discrimination from society, including nurses. This study aims to explore nurses’ perspectives on caring for HIV/AIDS-infected MSM. A descriptive qualitative design was utilized. Fifteen nurses who cared for HIV/AIDS-positive MSM in the two hospitals in Jakarta, Indonesia, were recruited with purposive sampling techniques. A semi-structured and in-depth interview was conducted. Data were analyzed using thematic analysis. We emerged three superordinate and nine subordinate themes: (1) negative nurse perceptions in the early phase of treatment, (2) nurse attitudes contrasting with negative perceptions, and (3) nurses with knowledge of HIV/AIDS. The negative perceptions appeared only at the beginning of the treatment phase, and thereafter, they were followed by a positive attitude. Nurses appeared to develop a better understanding after interacting with their patients and receiving training on HIV/AIDS. Therefore, intensive training is expected to not only increase their knowledge but to encourage a positive attitude.

## 1. Introduction

The incidence of the HIV epidemic in Indonesia is the highest among several critical populations. Based on statistics from the Indonesian Health Profile, the number of adult HIV-positive reports remained high [1]. These numbers can be contrasted to those from 2018 when 50,282 HIV-positive infections were recorded in Indonesia [2]. While HIV transmission rates among female sex workers and those who inject drugs have remained stable or declined, the incidence of HIV among men who have had sex with another man (MSM) has increased at least three-fold over the previous decade, going from 5.3% in 2007 to 17.9% in 2019. MSM accounts for roughly one-third of new infections in the region, with numerous nations facing a high and rising prevalence [3].

In Indonesia, HIV/AIDS-positive MSM avoid identification when they are sick and need care from health personnel, including nurses. This is because they often face stigmatization and discrimination. The stigma against HIV/AIDS-positive MSM increases in severity when coupled with the condition of HIV/AIDS and experiencing uncertainty such as constant suffering, fear, and anxiety in all phases of life [4]. Those impacted by HIV/AIDS tend to experience fear, ostracization, marginalization, intimidation, and hopelessness. This stigmatization not only comes from the environment but from health workers, including nurses, who may provide substandard care [5,6].

A study in the United States showed that the attitude explicitly (consciously) and implicitly (subconsciously) shown by nurses when picking treatments ignored the MSM person, certainly affecting the treatment provided [7]. This led to mistrust from HIV/AIDS-positive MSM of the health system and health care. In Indonesia, the incompetence of health care providers and nurses in implementing care can be biased because it is contrary to religion and culture [8]. This is due to the lack of studies and specific guidelines related to handling HIV/AIDS-positive MSM which results in a lack of knowledge, understanding, and skills of clinical nurses when working or dealing with MSM.

The participation of nurses in the development of HIV disease management strategies for and in collaboration with people directly impacted by the disease is necessary. Since the early 1980s, the HIV epidemic has disproportionately harmed the sexual orientation and gender identity of people—one of them is HIV/AIDS-positive MSM [9,10]. Based on the described problems, we aimed to conduct research that explored the perceptions of nurses regarding the nursing care of HIV/AIDS-positive MSM. This study presents the possibilities and challenges for nurses to connect nursing principles and practices with the emotional, psychological, spiritual, and sexual health needs of MSM and communities in a manner that respects human dignity.

## 2. Materials and Methods

### 2.1. Study Design

This study used a qualitative descriptive approach. This approach seeks to describe all situations and circumstances as they are, including what is still happening or being carried out at the time of the study [11].

### 2.2. Research Characteristics and Reflexivity

In this study, the researchers directly conducted semi-structured interviews. The researchers have a background as psychiatric nurse specialists. The research team was composed of men and women, and they all had international-level research and publication experience and training.

### 2.3. Participant and Setting

Participants in this study were nurses who cared for HIV/AIDS-positive MSM at a Government and Private Hospital in Indonesia. The sampling technique selected them using purposive sampling. Purposive sampling is the most recommended method for qualitative research because qualitative research requires closeness or intimacy as part of the criteria and requires logic for any decision made [12]. Participants were determined by the specific inclusion criteria and were nurses who cared for HIV/AIDS-positive MSM in the inpatient unit with at least a Diploma of Nursing level education. A key informant and nurse counselor in the two hospitals aided the process of recruiting researchers from those hospitals. The number of participants recruited in this study was 15 participants. The first government hospitals recruited as many as eight participants, and seven others came from private hospitals.

### 2.4. Ethical Considerations

This study received approval from the Ethics Committee of the Faculty of Nursing at the University of Indonesia with No. 153/UN2.F12.D/HKP.02.04/2018. This study followed the declaration of Helsinki [13]. Participants had the right to freely withdraw from the research. Participants could answer or not answer any question that made them feel uncomfortable. In addition, the researchers maintained their privacy throughout the interview. All recorded data were anonymized by coding according to P1-P15. The participants in this study were volunteers, and the study had no possibility of physically or mentally harming the participants.

### 2.5. Data Collection

The researchers used semi-structured and in-depth interviews to explore the nurse’s perspectives who were caring for HIV/AIDS-positive MSM at a Government and Private Hospital in Indonesia from May to July 2018. The interview activities were carried out directly by first obtaining informed consent and asking for permission to record their voice during the interview. The average interview time ranged from 30 to 77 min. The tools used in this research were the researcher as the lead instrument, tape recorders, mobile phones, semi-structured interview guidelines, and field notes. The interview began by building trust between the participants and interviewers. The interviews then proceeded with questions covering “ *Can you explain to me about HIV/AIDS-positive MSM and what do think about it?”*, “ *Can you tell me how you feel when you provide nursing care for HIV/AIDS-positive MSM?*”, “ *What are the problems you faced while caring for an HIV/AIDS-positive MSM?*” and *“what can you get from caring for HIV/AIDS-positive MSM?”*

### 2.6. Data Analysis

The NVIVO 12 software (QSR International) was used in this study to aid in the categorization and organization of the data collected through participant quotes and observations. The analysis of the interview results used a thematic approach. The stages consisted of (1) gaining familiarity with the data; (2) generating the initial codes; (3) searching for themes; (4) reviewing the themes; (5) defining and naming the themes; and (6) producing the report [14].

### 2.7. Triangulation and Trustworthiness

In addition, the researchers also conducted verification tests on the interview results of the participants. When the data reached saturation, the research process stopped. The triangulation in this study was the triangulation of sources, techniques, and research time [15]. Thus, the trustworthiness of the data in this study was verified by conducting credibility tests, undertaking member and self-reflexivity checks, and accomplishing transferability by adapting the results of the research to a thesis statement. Dependability acted upon by audit inquiry, thesis advisers involved as auditors, and conformability executed by attaching the end of the study report or article, rendered the investigator’s logic easier to follow for readers [11].

## 3. Results

### 3.1. Characteristic of Participants

The participants in this study were nurses working in the inpatient units of two hospitals, both government and private hospitals. Participants overall amounted to 15 individuals, composed of 11 female nurses and 4 male nurses. The age range of the participants was between 26 and 49 years. The education level of the participants varied: eight people graduated with a Diploma in Nursing, six people underwent the Undergraduate Program (Bachelor of Nursing), and one graduated from a professional (nursing) program with 3–20 years of experience in health care (See Table 1).

### 3.2. Themes

In this study, we emerged three superordinate themes, namely: (1) negative nurse perception in the early phase of treatment; (2) nurse attitudes contrasted with negative perceptions; and (3) nurses who have well knowledge of HIV/AIDS. Detailed information about superordinate and subordinate themes is explained below and presented in Table 2.

#### 3.2.1. Theme 1: Negative Nurse Perception in the Early Phase of Treatment

The first theme raised by researchers was negative perceptions from nurses in the early phases of nursing care, especially during their first experience caring for HIV/AIDS-positive MSM. In this theme, we raised four subordinate themes: (1) uncomfortable; (2) uncommon; (3) afraid; and (4) self-stigma.

##### Uncomfortable

Nurses expressed feelings of discomfort at the beginning of caring for HIV/AIDS-positive MSM. For them (nurses), it is strange and disgusting to have to deal with HIV/AIDS-positive MSM. (See quotes 1–2).

##### Uncommon

The nurses expressed something uncommon. At first, the nurses were surprised when they saw MSM dressed and behaving like a woman. This is very different from the teachings, beliefs, and culture they adhere to. (See quote 3)

##### Afraid

At first, the fear that arose from the nurse was due to a lack of knowledge. This can be seen as a fear of contracting from touch. (See quotes 4–5)

##### Self-Stigma

A lot of inaccurate information circulating in the community can lead to self-stigma among nurses. This can be seen in the behavior of nurses who are afraid of caring for their patients and who tend to avoid them. (See quote 6).

#### 3.2.2. Theme 2: Nurse Attitudes Contrasted with Negative Perceptions

The majority of participants showed a different attitude that contradicted the negative perceptions the others conveyed. Participants were accepting of the condition of MSM living with HIV. In this theme, we obtained three subordinate themes which constructed the superordinate theme.

##### Acceptance

Nurses who received sufficient knowledge regarding nursing care and training about caring for MSM patients living with HIV/AIDS showed an attitude of acceptance and willingness to provide care. (See quotes 7–8).

##### Sincerity and Empathy

Instinctively, the nurses’ empathy emerged when they were able to be close to and care for HIV/AIDS-positive MSM. (See quotes 9–10)

##### Professionalism

The nurses showed a professional attitude, such as their willingness to care for, provide protection, and provide nursing care for HIV/AIDS-positive MSM. It built a close relationship between the patient and the nurse. (See quotes 11–12)

#### 3.2.3. Theme 3: Nurses Have Well Knowledge of HIV/AIDS

##### HIV/AIDS Information and Treatment

The nurse stated that she obtained knowledge and information regarding the care of MSM patients living with HIV/AIDS from the hospital. The information and knowledge obtained are very useful to be able to provide better care for MSM patients living with HIV/AIDS. (See quotes 13–14).

##### Building a Relationship with Patient

Not only knowledge about care, but nurses also gained the experience to build a good relationship with HIV/AIDS-positive MSM. For nurses, building a good relationship between the nurses and patients can increase closeness and reduce the incidence of being lost to follow-up. (See quotes 9–10) (See quotes 15–16)

## 4. Discussion

This study found that nurses had a positive perception of MSM during HIV care, although at first, the nurses had a negative perception. On the other hand, a negative perception may negatively influence patient–provider interactions within the therapeutic setting. Additionally, a lack of provider training in sexual orientation and gender identity cultural competence may result in knowledge and negative perceptions. The findings of this study are discussed based on the following themes.

### 4.1. Negative Nurse Perception in Early Phase of Treatment

In this study, we found that nurses had a negative perception during implementation and nursing care to HIV/AIDS-positive MSM. There was a feeling of lamentation, shock, and confusion when dealing with them. Usually, these feelings occurred at the time they first took care of the person or in their early experiences caring for HIV/AIDS-positive MSM. Negative perceptions are perceptions that describe the knowledge and the response or responses that are inconsistent with the perceived object [16].

When providing care to patients, nurses feel uncomfortable because nurses have to care for patients with HIV/AIDS. This can happen to nurses who have just taken care of HIV/AIDS-positive MSM. This finding is in line with previous examples in Puerto Rico, which stated uncomfortable feelings when caring for HIV/AIDS-positive MSM [17]. In addition, nurses feel something different or uncommon when caring for HIV/AIDS-positive MSM. This can happen when nurses have never cared for patients with sexual and gender minorities such as MSM. In addition, it is also due to differences in opinions and views regarding the beliefs and culture adopted by nurses when it comes to caring for patients with sexual and gender minorities [17,18].

Furthermore, nurses expressed difficult feelings when treating HIV/AIDS-positive MSM because they considered persons with this condition to be a very sensitive subject. Another perception was the irrational fear that they would be infected with HIV/AIDS. Previous studies have also mentioned fear of treatment if infected with HIVD/AIDS [19,20]. However, this can be overcome with good knowledge. In addition, negative perceptions that arise can be caused by self-stigma from nurses. Self-stigma is where nurses provide stigma about themselves, that they will be infected if caring for HIV/AIDS patients [21]. Previous research has argued that self-stigma can be overcome with awareness and social empowerment [22].

### 4.2. Nurse Attitude Contrasted with Negative Perceptions

We found that nurses showed an attitude of acceptance when caring for HIV/AIDS-positive MSM. Nurses offered an open attitude and provided good care. This is because the perceptions of nurses who were initially negative due to lack of information or knowledge became positive after gaining knowledge and training along with the treatment process [23]. Perceptions, in general, are influenced by several factors, one of which is value. Value is a concept that is formed from life experiences with friends, culture, education, work, and relationships associated with religious beliefs, family ties, sexual preference, creed, and gender or ethnic group roles [24]. As a nurse, being able to understand the patient’s condition and recognize the feelings and problems experienced by the patient is a form of caring. In Indonesia, a culture of mutual respect is in accordance with the principles of caring in nursing. Nursing education in Indonesia emphasizes the importance of knowing the feelings and needs of the patients [25]. In this way, the problems experienced by patients can be solved. This finding is related to the previous study [26]

Empathy is compatible with the goal of nursing, to show sincerity and ease suffering, and as a form of professional intervention. When combined with the professional standards of nursing care, it assists in the provision of comfort and a person’s well-being [27,28]. Nurses must recognize and understand themselves to accept others and to understand the patient’s strengths and weaknesses, hopes, fears, desires, and needs. When they can accept the patients, they can experience the spectrum of affection: empathy, compassion, and altruism. The main purpose of the relationship between the patient and the nurse is the health, well-being, and comfort of the person [29,30].

The attitude of nurses—as opposed to their feelings when treating an MSM person as well as one of the themes of research in the United States related to the attitude expressed by nurses while caring for lesbian, gay, and transgender (LGT) persons—emerges as “ *open arms, the heart of the conflict*”; this means a nurse shows an open attitude with an LGT person and their condition, but it is contrary to their conscience [31]. This contrariness occurs because they conflict with professed religious beliefs. However, in practice, they attempt to maintain good posture, trying to resolve this inner conflict by providing the best care to maintain their professionalism as a nurse. Moreover, as a professional, nurses must have the skills and sensitivity to develop a therapeutic relationship with the person. By recognizing the value system, the nurse can identify the value systems’ conflict, be able to distinguish the values and goals of the person, and assist persons in choosing the care they require [32].

### 4.3. Nurse Have Well knowledge of HIV/AIDS

Nurses’ negative or positive perceptions of persons with HIV/AIDS related to their knowledge of HIV/AIDS [33]. Knowledge is the most important domain that informs one’s actions. There are certain factors that affect a person’s attitude, like a personal experience, the influence of others, the influence of culture, education, mass media, religious institutions, and one’s own emotions, and these factors are part of accrued knowledge [34]. Detailed knowledge is then obtained from various sources such as education, age, interests, experience, and work-life [34]. Higher education would teach a person to think more logically and rationally and also improve the cognitive skills of a person, which is necessary to be able to continue to learn [35]. In conclusion, the participants’ education in this study also correlated to their understanding, especially their understanding of the condition of HIV/AIDS-positive MSM; with higher education, the nurses tended to more readily acquire information and wider insight.

In addition, with good knowledge, nurses can establish good relationships with patients. That way, HIV/AIDS-positive MSM can have a good support system. Previous studies have shown that good relationships and support from both nurses and peers can improve the quality of life of HIV/AIDS-positive MSM and reduce loss to follow-up rates [36,37,38].

### 4.4. Implication to Clinical Practice

Based on the interviews performed by researchers with participants in the two hospitals, the nurses had sound knowledge regarding the transmission and treatment of HIV/AIDS in HIV/AIDS-positive MSM with the condition. This aligns with research in Indonesia which states that the level of knowledge nurses have in relation to HIV/AIDS as a whole is sufficient [39]. The statement matches those of several participants who mentioned that they must update their knowledge. It can be concluded that the higher the knowledge and skills possessed, the better nursing care is given. There should be mandatory training in affirming and competent treatment for sexual orientation and gender identity patients, as recommended by the Joint Commission for the Accreditation of Healthcare Organizations (JCAHO) [40]. From the results of this study, an initial understanding regarding the care of patients with HIV/AIDS is needed for new nurses to avoid negative perceptions such as discrimination and stigma.

### 4.5. Study Limitation

In this study, we have some limitations, such as (1) the study is limited to nurses about their perception while caring for MSM living with HIV. This can lead to different perceptions for readers. In addition, information related to the perceptions of HIV/AIDS-positive MSM on the quality of care provided by nurses or other health workers can also be considered. Then, (2) this study is limited to one research area with two data collection location settings. Sampling from several regions can provide a more varied and informative description and information related to the perceptions of nurses in caring for HIV/AIDS-positive MSM in terms of culture, beliefs, and others.

## 5. Conclusions

The negative perception appeared only at the beginning of the treatment phase, thereafter followed in a positive manner. This may occur due to the lack of knowledge or training of nurses. With good knowledge, nurses can provide comprehensive nursing care and build good relationships between nurses and patients. This is very necessary to increase closeness, improve recovery, and prevent loss to follow-up events. The results of this study can be used as a source of information for nurses, especially those who care for patients with HIV/AIDS. In addition, the introduction, orientation, and training for new nurses who will care for patients with HIV/AIDS are needed to avoid negative perceptions.

## Figures and Tables

**Table 1:** Characteristics of participants.

Number	Age (Years)	Biological Gender	Religion	Education	Work Tenure with MSM Person
P1	26	Female	Islam	Diploma	6 years
P2	40	Female	Islam	Diploma	8 years
P3	44	Female	Islam	Undergraduate	5 years
P4	42	Male	Islam	Undergraduate	N/I
P5	35	Female	Islam	Undergraduate	N/I
P6	38	Male	Islam	Undergraduate	N/I
P7	38	Male	Islam	Undergraduate	3 years
P8	46	Female	Catholic	Undergraduate	26 years
P9	26	Female	Islam	Diploma	5 years
P10	32	Female	Islam	Undergraduate	8 years
P11	40	Male	Islam	RN program	N/I
P12	43	Female	Islam	Diploma	N/I
P13	49	Female	Hindu	Diploma	N/I
P14	48	Female	Catholic	Diploma	N/I
P15	42	Female	Catholic	Diploma	N/I

Note. Not information (N/I); Registered Nurse (RN).

**Table 2:** Superordinate, subordinate themes, and participant quotations.

Superordinate Themes	Subordinate Themes	Quotations
Negative nurse perception in the early phase of treatment	Uncomfortable	Q1: “Honestly, I was surprised, he looks handsome but how come he is MSM living with HIV. At first, I felt strange and uncomfortable...” (P1)
		Q2: “After hearing the story I felt uncomfortable, and it turned out that there were a lot of MSM around me. To be honest, I felt uncomfortable” (P4)
	Uncommon	Q3: “For me personally, that is very strange and inappropriate. Men dress like women, dress up, and even behave like women. Personally, this is very uncommon...” (P8)
	Afraid	Q4: “For me, it was very strange. At first, I was very scared; there was even MSM who once teased me... hahaha (laughs). Besides that, I did not want to be close to them, I was afraid that I might get infected...” (P14)
		Q5: “When conducting examinations, I always use double protection. I am very afraid if I get infected. In fact, I try to avoid coming in contact with them as much as possible” (P2)
	Self-stigma	Q6: “Before getting information and training regarding the care of patients with HIV, I received information that actually directed me to self-stigmatism where I was afraid and tended to avoid HIV/AIDS-positive MSM”
Nurse attitudes contrasted with negative perceptions	Acceptance	Q7: “After understanding everything, I started to accept them (HIV/AIDS-positive MSM). They are all very kind and cared for each another…” (P3)
		Q8: “mmmm… (mumbling), I think… no one wants to be in their position (HIV/AIDS-positive MSM). They are already different in society, suffer a lot of discrimination, are seen as different, even looked down upon. Apart from that, they also suffer HIV/AIDS. Maybe they are wrong, but even if they are in the hospital, they still get bad treatment (discrimination), how bad I am. Personally, I accept their condition, I take care of them. They are actually good people.” (P15)
	Sincerity and empathy	Q9: “Sometimes, I feel sorry for their destiny…, but what should I do? Sometimes, I feel sad when I hear their stories. Being shunned by their families and society…” (P8)
		Q10: “I am sincere in taking care of them, how pity they are... if not us (nurses), who else? Whether they will return to their nature or not, let that be between him (HIV/AIDS-positive MSM) and God” (P14)
	Professionalism	Q11: “I have to be professional... I cannot differentiate one patient (HIV/AIDS-positive MSM) and each other. Maybe it’s my destiny to work as a nurse who treats patients like that (HIV/AIDS-positive MSM) (P3)
		Q12: “As a nurse, I have to protect myself and provide protection for them (HIV/AIDS-positive MSM) while they are hospitalized. Not only that, gaining their trust is very important and you have to be able to maintain that trust” (P13)
Nurses have well knowledge of HIV/AIDS	HIV/AIDS information and treatment	Q13: “Personally, I would like to thank the hospital, because given me the opportunity to gain comprehensive knowledge, especially in the care of HIV/AIDS patients. Of course, we do not only treat them physically, but also psychologically...” (P8)
		Q14: “Of course… we can treat well, the medicines needed, and the control schedule. In fact, I am now a nurse counselor for HIV/AIDS-positive MSM. It requires sufficient knowledge and information to help these patients (HIV/AIDS-positive MSM).” (P10)
	Building relationship with patient	Q15: “We take care of him physically as well as psychologically… of course to be able to take care of him psychologically, the ability to build relationships is really needed…” (P7)
		Q16: “We (nurses) and the patients (HIV/AIDS-positive MSM) are now like family… we always joke together; they even often bring us food. Most importantly, they are happy with the treatment I provide.” (P1)

## Data Availability

Not applicable.
